# Upregulation of IFNE in cervical biopsies of patients with high‐risk human papillomavirus infections

**DOI:** 10.1002/iid3.1111

**Published:** 2023-12-06

**Authors:** Lu Zhang, Yuechen Zhou, Xiaoyan Xing, Hua Li, Zhan Zhang, Ruiya Qian, Xiaoyu Hu

**Affiliations:** ^1^ Department of Gynecology, Beijing Obstetrics and Gynecology Hospital Capital Medical University Beijing China; ^2^ Beijing Maternal and Child Health Care Hospital Beijing China; ^3^ School of Medicine Tsinghua University Beijing China; ^4^ Institute for Immunology and School of Medicine Tsinghua University Beijing China; ^5^ Tsinghua‐Peking Center for Life Sciences Beijing China; ^6^ Beijing Key Laboratory for Immunological Research on Chronic Diseases Beijing China

**Keywords:** cervical epithelial cells, female reproductive tract, human papillomavirus, interferon epsilon

## Abstract

**Problem:**

Interferon epsilon (IFN‐ε) is constitutively expressed in the epithelium of female reproductive tract and confers vital protection against sexually transmitted pathogens in mouse models. However, there is limited insight into the role of IFN‐ε in human sexually transmitted infections such as human papillomavirus (HPV).

**Method of Study:**

Cervical biopsies were obtained from high‐risk (HR) HPV positive (*n* = 28) and HR‐HPV negative (*n* = 10) women. mRNA expression of IFN‐ε in cervical tissues was measured by qPCR. Expression of the IFN‐ε protein was determined by Western blot analysis, immunohistochemistry and immunofluorescence staining.

**Results:**

mRNA expression of IFN‐ε was higher in the ectocervix than that of other IFNs, and was further upregulated in HR‐HPV positive women compared with HR‐HPV negative women. Expression of the IFN‐ε protein was comparable between HR‐HPV infected patients and healthy controls.

**Conclusions:**

These results reveal differential expression of IFN‐ε mRNA between individuals with or without HR‐HPV infection, and imply direct or indirect regulatory mechanisms for IFN‐ε transcription by HPV. Expression of IFN‐ε protein in HPV infections would require further validation.

## Graphical abstract

The role of interferon epsilon (IFN‐ε) in human sexually transmitted infections, particularly human papillomavirus (HPV), remains poorly understood. This study examined the expression of IFN‐ε in cervical tissues of women with high‐risk HPV (HR‐HPV) infection. The results showed increased mRNA expression of IFN‐ε in HR‐HPV‐positive women compared to HR‐HPV‐negative women.

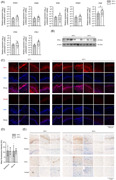

## 1 INTRODUCTION

The recently characterized interferon (IFN)‐ε is unique for its tissue‐specific expression profile. Unlike other type I IFNs, IFN‐ε is constitutively expressed in the epithelial cells of the female reproductive tract (FRT).^1^, ^2^ This unique feature suggests its potential role in protecting against sexually transmitted infections (STIs) in the FRT, as demonstrated in murine models.^1^ IFN‐ε binds to IFNα/β receptor 1 and 2 (IFNAR1 and IFNAR2) and regulates expression of IFN‐stimulated genes (ISGs) via the JAK‐STAT signaling pathway.^1^, ^3^ Thus, it appears to share the antiviral, antibacterial, antiproliferative and immunoregulatory properties with other type I IFNs. *In vitro* investigations into these biological functions mostly showed subdued potency when compared to other IFNs.^4^, ^5^ For example, antiviral activity against Semliki Forest virus (SFV) of IFN‐ε in vitro is 100 and 1000‐fold weaker than that of IFN‐α and IFN‐β, respectively. Similarly, its antibacterial activity is diminished 1000‐fold compared to IFN‐β.^5^ IFN‐ε is reported to have comparable potency of HIV restriction factor induction as conventional type I IFNs.^6^ *In vivo* studies have shown that IFN‐ε deficiency in mice is associated with increased susceptibility and worsened disease outcomes in herpes simplex virus type 2 (HSV‐2) and *Chlamydia* infection.^1^

In contrast to most type I interferons (IFNs), which are highly inducible by pathogens,^7^ *Ifne* expression remains unaltered in mice following infection with Mengo virus,^8^ *Chlamydia muridarum*, and HSV‐2.^1^ However, a pilot study by Nickodem et al. reported downregulation of IFN‐ε in HSV‐infected women in pregnancy,^9^ suggesting a potential regulation of IFN‐ε expression by certain pathogens in human. Characterization of human type I IFN locus revealed that *IFNE* has a highly conserved 5′ flanking region sharing >70% identity with its counterpart in mice.^10^ Previous attempts at characterizing putative transcription factor binding sites in the 5′ flanking region of IFN‐ε genes have yielded contradictory results regarding the presence of response elements to pattern recognition receptor (PRR) pathways. Recent analysis of the ~1 kb 5′ flanking region by Fung et al.^1^ showed that *Ifne* lacks these response elements, which contradicts earlier reports.^10^ This observation is supported by in vitro evidence, such as the limited impact on IFN‐ε expression in HeLa cells when stimulated with PRR ligands like poly(I:C). Additionally, IFN regulatory factor (IRF)‐3, −5, and −7 expression vectors fail to induce IFN‐ε expression in luciferase reporter assays.^1^, ^11^ Subsequent research has consequently shifted its focus towards the hormonal aspect of IFN‐ε regulation. A notable discovery in this regard was the lack of correlation between progesterone receptor and IFN‐ε expression in the FRT epithelium. Sex hormones are dominant regulators of FRT physiology and exert a profound impact on immune responses against STIs. Unique expression profiles of IFN‐ε add another layer of complexity to its host defense function.

While many fundamental questions regarding the function and regulation of IFN‐ε remain to be fully understood, previous studies using cell lines and mouse models collectively suggest that IFN‐ε may provide a mild but persistent level of protection against pathogens specifically in the FRT mucosa. Human papillomaviruses (HPVs) are among the most prevalent sexually transmitted pathogens affecting the FRT. HPVs are highly tissue‐tropic viruses that infect stratified epithelia, especially that of the human cervix.^12^ HPVs have received great public attention due to the oncogenic effects of certain subtypes. Oncogenic or high‐risk (HR) HPV types are responsible for approximately 4.5% of all cancers worldwide, including the majority of cervical cancer and a substantial portion of other anogenital and oropharyngeal cancers.^13^ While most HPV infections are cleared by the immune system, persistent infections of HR‐HPV in the basal layers of epithelial cells can lead to the development of precancerous lesion or cancer. Multivalent prophylactic HPV vaccines have proven to be highly effective in preventing HPV‐attributable cancers, but they do not eliminate pre‐existing infections.^14^ Further investigations into the viral‐host interactions are necessary to develop therapeutics against persistent HR‐HPV infections. The benefits of type I IFNs in pathogen control have been extensively studied. Given its unique spatial expression patterns, IFN‐ε holds the potential to be a pivotal component in immune responses during HPV infections.

Given the limited number of clinical studies, there is still much to uncover regarding the roles of IFN‐ε in human. Particularly, IFN‐ε expression in patients infected with sexually‐transmitted pathogens, such as HPV, is poorly characterized. Marrero‐Rodriguez et al.^15^ reported that IFN‐ε expression is elevated in cervical cancer, but it remains unclear whether IFN‐ε expression is regulated by HPV infection. To answer this question, we investigated the expression of IFN‐ε at both the mRNA and protein levels in women with or without HR‐HPV infections.

## 2 MATERIALS AND METHODS

### 2.1 Patient samples

This study was approved by the ethics committee of Beijing Obstetrics and Gynecology Hospital, Capital Medical University (2019‐KY‐079‐02). A total of 38 women (28 HR‐HPV positive and 10 HR‐HPV negative) were recruited from Beijing Obstetrics and Gynecology Hospital and Beijing Chaoyang Integrative Medicine Emergency Medical Center and signed informed consent before study participation. Samples were obtained during either routine cervical biopsy procedures for diagnosis of precancerous conditions and cervical cancer, or surgeries for nonmalignant gynecological diseases. Cervical lesions were detected with ThinPrep cytologic test before sample collection. Diagnosis was confirmed histologically in patients eligible for cervical biopsy. Cervical tissues (3 × 3 × 3 mm^3^) obtained from HR‐HPV negative individuals were divided into two aliquots. One aliquot was frozen in liquid nitrogen and stored in −80℃ before lysis using TRIzol (Invitrogen). The other was incubated in 4% paraformaldehyde or 10% formalin for 24 h at 4℃. Fixed samples were later transferred to 70% EtOH. Due to the limited volume of samples obtained from HR‐HPV positive patients, only a subset of these samples (*n* = 10) were divided into two aliquots for both mRNA and histology analysis. The remaining samples were either immediately frozen in liquid nitrogen (*n* = 12) or fixed in 4% paraformaldehyde or 10% formalin (*n* = 6).

### 2.2 **Quantitative polymerase chain reaction** (qPCR)

RNA was extracted from samples with TRIzol reagent (Invitrogen) and treated with TURBO DNase (Invitrogen). cDNA was synthesized using M‐MLV reverse transcriptase (Takara) and oligo (dT) primers. qPCR was performed using PowerUp SYBR Green master mix (Applied Biosystems) on the StepOnePlus system (Applied Biosystems). Expression of target genes was normalized to housekeeping gene *EEF1A1* with the comparative threshold cycle method. Primer sequences are listed in Table 1.

**Table 1:** qPCR primers.

Gene	Forward (5′–3′)	Reverse (5′–3′)
*EEF1A1*	GGACACGTAGATTCGGGCAA	AGGAGCCCTTTCCCATCTCA
*IFNA2*	TTCGTATGCCAGCTCACCTT	GGACTAGTGCCTTAAGAGCTGA
*IFNB1*	TCTCCTGTTGTGCTTCTCCAC	GCCTCCCATTCAATTGCCAC
*IFNE*	CAGCCGATGTCTGTTCTTTGTG	CCTCTAGTCCCTCCACCTACC
*IFNK*	GTCCCTACAGGCCTTCAACA	GGTTCAGGTACTCTGCTTGCT
*IFNW1*	TCTACTGGCAGCCCTAGTGA	CTGGGGGAACCTGAAGTCTC
*IFNG*	ACTGCCAGGACCCATATGTAAA	TTTTCTGTCACTCTCCTCTTTCC
*IFNL1*	ATATGTGGCCGATGGGAACC	GTCAGGGCTGCAGCTTCATA

### 2.3 Western blot analysis

Total proteins were extracted with TRIzol reagent (Invitrogen) and dissolved in 1% sodium dodecyl sulfate (SDS). For western blot analysis, proteins were separated by 10% SDS polyacrylamide gel electrophoresis and transferred to a polyvinylidene fluoride membrane (Millipore). After blocking with 2.5% BSA, membranes were incubated at 4℃ for 24 h with IFN‐ε mouse antibody (1:1000, Novus Biologicals) or β‐actin antibody (1:1000, ABclonal). The membranes were then washed and incubated with goat antimouse IgG (H&L)‐HRP conjugated antibody (1:10000, Gene‐Protein Link) or goat antirabbit IgG (H&L)‐HRP conjugated antibody (1:10000, Gene‐Protein Link). Proteins were visualized with Immobilon Western chemiluminescent HRP substrate (Millipore).

### 2.4 Immunohistochemistry

For each fixed and paraffin‐embedded tissue, 5‐µM sections were cut and adhered to silanized slides. Heat induced antigen retrieval was performed using antigen retrieval buffer (10 mM Tris base, 1 mM EDTA solution, 0.05% Tween 20, pH 9.0) for 20 min after hydration. Sections were blocked with hydrogen peroxide blocking buffer (Zhongshan Golden Bridge) for 5 min and 10% goat serum in 0.01% Tween‐PBS for 30 min at room temperature, then incubated with IFN‐ε antibody (1 µg/mL, Novus Biologicals) or mouse IgG2b (1 µg/mL, BioLengends) as isotype‐negative control, both diluted with antigen dilution buffer (Zhongshan Golden Bridge), for 24 h at 4℃. Sections were washed with 0.01% Tween‐PBS for 2 h and incubated with goat antimouse IgG (H&L)‐HRP conjugated antibody (1:100, Gene‐Protein Link) for 1 h at room temperature. Sections were washed again with 0.01% Tween‐PBS for 45 min and stained using the enhanced HRP‐DAB Chromogenic Kit (TIANGEN). Sections were then counterstained with hematoxylin (Solarbio), dehydrated, and mounted in neutral balsam.

### 2.5 Immunofluorescence microscopy and quantification

For immunofluorescence, sections were blocked with 10% goat serum and incubated with IFN‐ε antibody (1 µg/mL, Novus biologicals) or mouse IgG2b (1 µg/mL, BioLengends) for 24 h at 4℃. Sections were then washed with 0.01% Tween‐PBS and incubated with antimouse IgG (Alexa Fluor 594 conjugate, 1:50, Cell Signaling Technology) for 2 h and DAPI for 5 min at room temperature. Sections were washed with H_2_O before mounting with antifade fluorescence mounting medium (Abcam). Mean fluorescence intensity of epithelium and stroma was quantified using ImageJ2 (v2.3.0/1.53f).

### 2.6 Statistical analysis

Statistical analysis was performed with two‐tailed Welch's *t*‐test or other tests where indicated. *p* < .05 was taken as statistically significant. Statistical analysis was performed using GraphPad Prism 9.1.1.

## 3 RESULTS

Cervical tissues were collected from a total of 38 enrolled individuals (10 negative for HR‐HPV and 28 positive for HR‐HPV). Due to the limited volume of each sample, only a subset of samples (10 h‐HPV negative and 22 h‐HPV positive) were subjected to RNA extraction and characterization of IFN expression at the mRNA level (demographic and clinical characteristics shown in Table 2).

**Table 2:** Statistical correlation between IFN‐ε expression and clinical characteristics.

Characteristics	Overall distribution of characteristics^a^	Comparison of IFN‐ε by characteristics (*p* value)
Age	40.35 ± 10.16	.43
<40	17 (0.53)	
≥40	15 (0.47)	
Hormonal status		.12^b^
Follicular stage	12 (0.38)	
Luteal stage	13 (0.41)	
Postmenopausal	3 (0.09)	
Postpartum	1 (0.03)	
Undergoing GnRH‐α therapy	3 (0.09)	
Gravidity	2.16 ± 1.27	.10
≤2	21 (0.66)	
>2	11 (0.34)	
Parity	1.00 ± 0.80	.51
0	9 (0.28)	
≥1	23 (0.72)	
HPV infection		.03^c^
HR‐HPV−	10 (0.31)	
HR‐HPV+	22 (0.69)	
Diagnosis		.15
SIL	14 (0.44)	
Non‐SIL	18 (0.56)	

^a^ Presented as average ± standard deviation or frequency (percent).

^b^ Data analyzed with the Brown‐Forsythe test.

^c^ Statistical significance.

**Figure 1:**
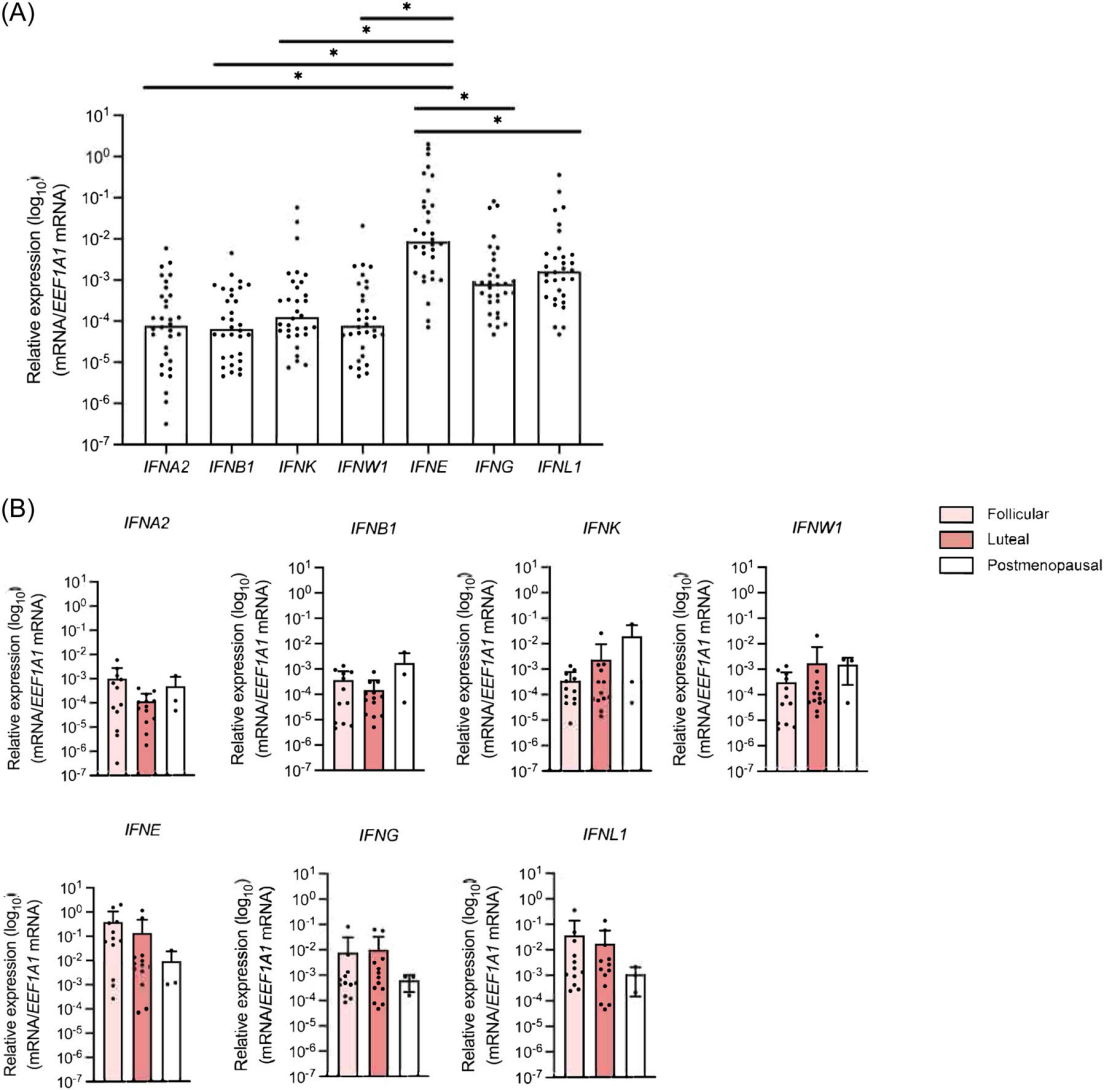
IFN‐ε is expressed at high levels in cervical tissue regardless of hormonal status. (Α) Cervical biopsy samples (*n* = 32) were examined for mRNA levels of IFN‐α2, IFN‐β, IFN‐ε, IFN‐κ, IFN‐ω, IFN‐γ, and IFN‐λ1. (B) IFN expression levels in cervical samples from patients in follicular (*n* = 12) and luteal (*n* = 13) stage and postmenopausal patients (*n* = 3). IFN expression of postpartum patients and patients undergoing GnRH‐α therapy is not shown.

To assess the expression of IFN‐ε in relation to other IFNs, we measured mRNA expression of type I IFNs (IFN‐α2, ‐β, ‐κ, ‐ω), type II IFN (IFN‐γ), and type III (IFN IFN‐λ1) alongside IFN‐ε. Our analysis revealed considerable variability in the expression levels of all IFN genes. Notably, *IFNE* showed higher expression than genes which encode other IFNs (Figure 1A), as previously documented.^2^ No significant difference were observed in *IFNE* expression among patients grouped by age (*p* = .43), gravidity (*p* = .10), parity (*p* = .51), or cervical squamous intraepithelial lesion (SIL) diagnosis (*p* = .15). The expression of *IFNE* and other genes encoding IFNs did not differ significantly among individuals at different stages of menstrual cycle (*p* = .12; Figure 1B), which was consistent with the observations of Bourke et al.^2^ in their studies with an American cohort.

**Figure 2:**
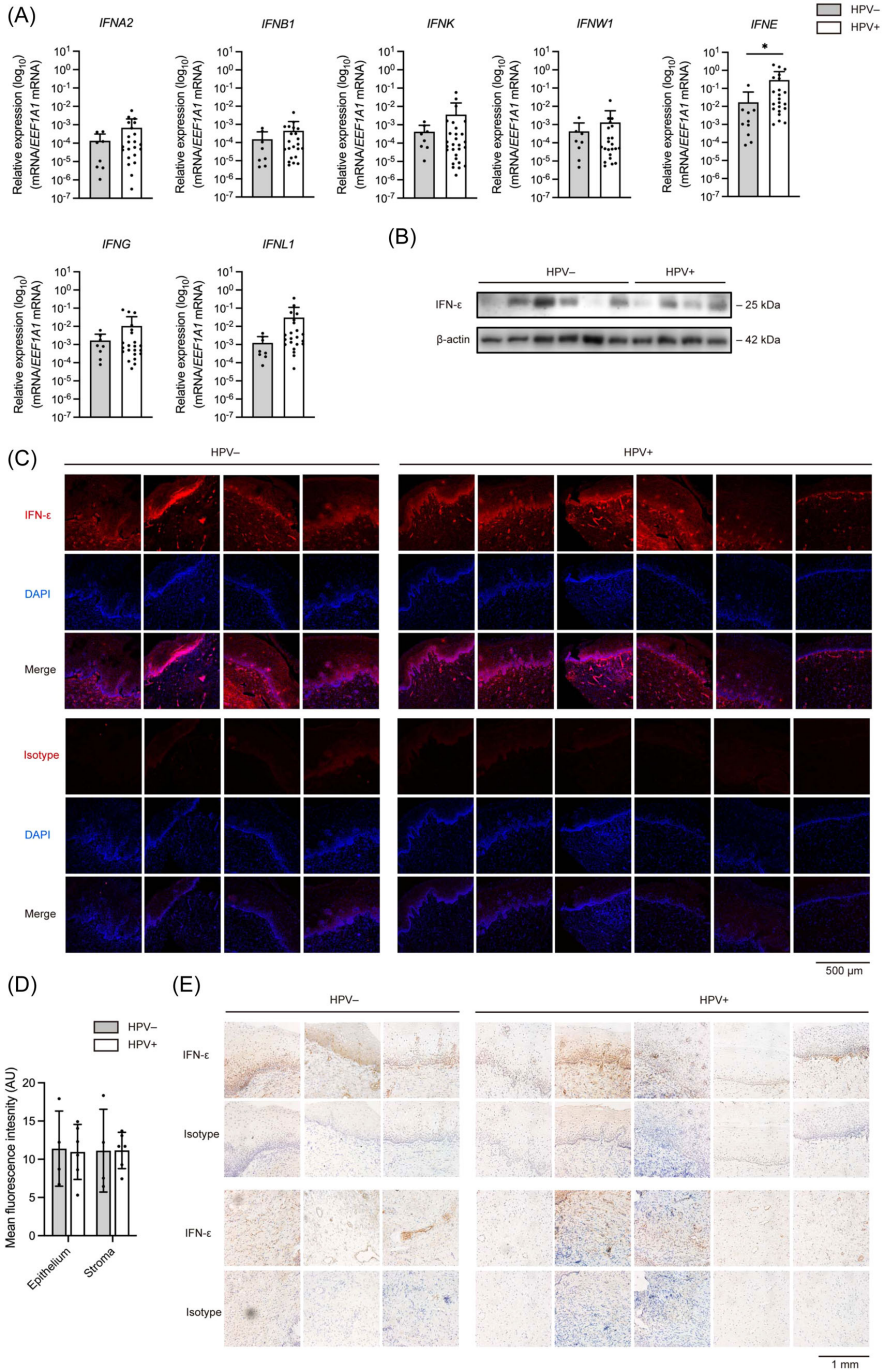
IFN‐ε expression is upregulated in HR‐HPV positive patients at the mRNA but not protein level. (A) IFN expression levels in cervical samples from HR‐HPV negative (*n* = 10) and HR‐HPV positive (*n* = 22) patients (*p < .05). (B) IFN‐ε expression determined in representative samples of HR‐HPV negative (*n* = 10) and HR‐HPV positive (*n* = 22) patients by immunoblotting. (C) Immunofluorescence staining for IFN‐ε (red) and DAPI (blue) in cervical tissues of HR‐HPV negative and HR‐HPV positive patients. (D) Quantitative analysis of immunofluorescence staining for IFN‐ε in cervical samples of HR‐HPV negative (*n* = 4) and HR‐HPV positive (*n* = 6) patients. (E) Immunohistochemical analysis of IFN‐ε protein levels in cervical biopsies of HR‐HPV positive and HR‐HPV negative patients.

Next, we examined IFN mRNA levels in HR‐HPV positive patients and healthy controls. HR‐HPV positive patients showed higher expression of *IFNE* compare to healthy individuals (*p* = .03), while no statistically significant differences were detected for the expression of other IFN genes between these two groups (Figure 2A).

IFN‐ε expression showed considerable variations at the protein level among individuals in both HR‐HPV positive and HR‐HPV negative groups. However, no substantial difference was observed between these two groups (Figure 2B). This prompted us to explore potential differences in the spatial expression patterns of IFN‐ε. Consistent with previous reports,^2^ immunofluorescence and immunohistochemical staining both demonstrated that IFN‐ε is primarily expressed in the basal layer of cervical epithelium (Figures 2C,E). We also observed strong signals in the stromal endothelial cells, which accounted for the unexpectedly high expression level of IFN‐ε in the stroma. Quantitative analysis of immunofluorescence staining demonstrated comparable expression between HR‐HPV positive and negative individuals (Figure 2D).

## 4 DISCUSSION

The critical role of IFNs in innate immune responses against viral infection has been well‐established. As a novel type I IFN predominantly found in FRT epithelium, IFN‐ε may confer vital protection against sexually transmitted pathogens. Bourke et al.^2^ have provided a comprehensive analysis of IFN‐ε expression at homeostasis, highlighting hormonal control in different anatomical locations of the FRT. However, IFN‐ε expression during infection remains to be elucidated. This study is the first to compare the expression of IFNs in HR‐HPV positive and HR‐HPV negative individuals. *IFNE* showed highest basal level of transcription among genes encoding IFNs and was further induced in HR‐HPV infection, suggesting a potential role in persistent protection. By contrast, other IFNs investigated in this study showed comparable mRNA levels in individuals with or without HR‐HPV infection.

Marrero‐Rodriguez et al.^15^ previously reported that IFN‐ε expression was upregulated in cervical cancer, and suggested that IFN‐ε could serve as a biomarker for cervical cancer. Here, we quantitatively assessed *IFNE* mRNA abundance in HR‐HPV infection and further showed that IFN‐ε is upregulated in HR‐HPV infected normal or precancerous tissue, suggesting a potential role for IFN‐ε in the host defense against HPV during early stages of the disease.

We did not observe significant differences in IFN‐ε protein abundance between the HR‐HPV positive and negative samples. Such discrepancies between the mRNA and protein abundance could be attributed to sample variability, particularly when working with a limited sample size, as in some cases the sample volume did not permit us to assess both protein and mRNA levels. Alternatively, this could suggest underlying regulatory mechanisms operating at the translational or posttranslational level. Future in‐depth mechanistic studies are warranted to gain a comprehensive understanding of these discrepancies and to unravel the precise mechanisms regulating IFN‐ε expression.

The specific mechanisms underlying the induction of IFN‐ε upon HR‐HPV infection remain elusive, but it is reasonable to speculate that both direct and indirect pathways may be at play. Experimental evidence has demonstrated that viral oncoproteins E5, E6 and E7 of certain HR‐HPV subtypes contribute to the immune evasion mechanisms of HR‐HPV by directly interfering with the IFN signaling cascade.^16^ These subtype‐specific regulations have been predominantly explored in the context of the expression of other type I IFNs, including IFN‐α, ‐β and ‐κ. HPV oncoproteins can act through inhibition of the PRR pathways.^17^, ^18^, ^19^, ^20^ Additionally, other mechanisms such as epigenetic silencing of IFN can also be involved.^21^ We were unable to examine subtype‐specific regulations on IFN expression due to limited sample size. Whether similar subtype‐specific modulatory pathways exist for IFN‐ε requires further investigation.

Induction of IFN‐α and ‐β in viral infections is dependent on pathogen sensing by cell‐surface and intracellular PRRs.^22^ Previous studies indicate that IFN‐ε is unlikely to be subject to such regulations.^3^ Yet, it remains uncertain whether IFN‐ε could be upregulated as a secondary effect to viral entry by cytokines and other immunoregulatory molecules in the tissue microenvironment or via interactions with other immune or nonimmune cell types. Reports have suggested that treatment with TNF‐α upregulates IFN‐ε expression by modulating mRNA stability,^11^ suggesting the potential involvement of multiple regulatory mechanisms operating at different levels.

This study also investigates the effects of other potential regulators, including sex hormones. Some of our observations were consistent with previous studies. *IFNE* expression did not demonstrate marked fluctuations in different stages of the menstrual cycle. There is a substantial amount of evidence indicating that mucosal immunity of the FRT is heavily regulated by sex hormones to avoid compromising conditions for fertilization and pregnancy.^23^ Bourke et al.^2^ first demonstrated the absence of hormonal regulation in IFN‐ε expression in the vagina and ectocervix and attributed this to the lack of progesterone receptors in the lower FRT. We further showed that there was no significant difference nor consistent trends in the expression of other IFNs at different stages of the menstrual cycle.

Intriguingly, we noticed strong IFN‐ε signals from the endothelial cells of cervical stroma in both immunofluorescence and immunohistochemical staining, which had largely been overlooked in previous characterizations of IFN‐ε expression.^2^ It remains to be determined whether these IFN‐ε^high^ endothelial cells are vascular or lymphatic. Cytokine secretion is considered to be among the many innate immune functions that endothelial cells share with macrophages.^24^, ^25^ High IFN‐ε expression potentially provides new evidence for the role of endothelial cells in protection against STIs.

Due to the cross‐sectional nature of this study, the clinical significance of IFN‐ε expression awaits future investigations. IFN‐ε has been proposed as a promising therapeutic target for mucosal viral infections, given its protective capacity and unique expression profile.^26^ Early clinical experiments with IFN‐α and ‐β treatment against HPV infection and associated diseases yielded inconsistent results.^27^ Unfavorable clinical outcomes may, in part, be explained by the detrimental effects of conventional IFNs in persistent viral infections, which are mostly illustrated in human immunodeficiency virus (HIV) and lymphocytic choriomeningitis virus infections.^26^ Less is understood regarding the role of IFN‐ε in chronic infections. Further follow‐up studies are warranted to establish a relation between IFN‐ε expression and clinical outcome.

## AUTHOR CONTRIBUTIONS

**Lu Zhang**: Conceptualization; Data curation; Formal analysis; Investigation; Methodology; Resources; Visualization; Writing—original draft; Writing—review & editing. **Yuechen Zhou**: Conceptualization; Formal analysis; Investigation; Methodology; Visualization; Writing—original draft; Writing—review & editing. **Xiaoyan Xing**: Conceptualization; Formal analysis; Investigation; Methodology; Visualization; Writing—original draft; Writing—review & editing. **Hua Li**: Data curation; Resources; Writing—review & editing. **Zhan Zhang**: Data curation; Resources; Writing—review & editing. **Ruiya Qian**: Data curation; Project administration; Resources; Writing—review & editing. **Xiaoyu Hu**: Conceptualization; Formal analysis; Funding acquisition; Investigation; Methodology; Project administration; Resources; Supervision; Visualization; Writing—original draft; Writing—review & editing.

## CONFLICT OF INTEREST STATEMENT

The authors declare that they have no known competing financial interests or personal relationships that could have appeared to influence the work reported in this paper.

## ETHICS STATEMENT

The authors confirm that the ethical policies of the journal, as noted on the journal's author guidelines page, have been adhered to and the appropriate ethical review committee approval has been received. The study conformed to the US Federal Policy for the Protection of Human Subjects.

## Data Availability

The authors confirm that the data supporting the findings of this study are available within the article.
